# Effects of Polypharmacy on Adverse Drug Reactions among Geriatric Outpatients at a Tertiary Care Hospital in Karachi: A Prospective Cohort Study

**DOI:** 10.1371/journal.pone.0112133

**Published:** 2014-11-17

**Authors:** Bilal Ahmed, Kashmira Nanji, Rakshinda Mujeeb, Muhammad Junaid Patel

**Affiliations:** 1 Department of Medicine, Aga Khan University, Karachi, Pakistan; 2 Department of Family Medicine, Aga Khan University, Karachi, Pakistan; David Geffen School of Medicine, United States of America

## Abstract

**Background:**

Adverse drug reactions (ADRs) present a challenging and expensive public health problem. Polypharmacy is defined according to the WHO criteria as the, “concurrent use of five or more different prescription medication”. Elderly are more prone to adverse reactions due to comorbid conditions, longer lists of medications and sensitivity to drug effects. The aim of the study is to estimate the incidence and strength of association of ADRs due to polypharmacy among the geriatric cohort attending outpatient clinics at a tertiary care center.

**Methods:**

A hospital based prospective cohort study was conducted at ambulatory care clinics of Aga Khan University Hospital April 2012 to March 2013. One thousand geriatrics patients (age ≥65 years) visiting ambulatory clinics were identified. They were divided on the basis of exposure (polypharmacy vs. no polypharmacy). We followed them from the time of their enrollment (day zero) to six weeks, checking up on them once a week. Incidence was calculated and Cox Proportional Hazard Model estimates were used.

**Results:**

The final analysis was performed on 1000 elderly patients. The occurrence of polypharmacy was 70% and the incidence of ADRs was 10.5% among the study cohort. The majority (30%) of patients were unable to read or write. The use of herbal medicine was reported by 3.2% of the patients and homeopathic by 3%. Our Cox adjusted model shows that polypharmacy was 2.3 times more associated with ADRs, con-current complementary and alternative medicine (CAM) was 7.4 times and those who cannot read and write were 1.5 times more associated with ADRs.

**Conclusion:**

The incidence of ADRs due to poly pharmacy is alarmingly high. The factors associated with ADRs are modifiable. Policies are needed to design and strengthen the prescription pattern.

## Background

Epidemiologic transition over the years has increased the percentage of elderly (aged greater than 65 years). The elderly people now constitute more than 60% of the world population, which in turn increases their hospital visits leading to multiple medications’ use [1]. Approximately there are 841 million elderly (60 years and older) people in the world. By 2050, nearly 8 in 10 of the world’s older population will be living in the less developed areas [2]. This increase in life expectancy has brought about increased numbers of certain chronic illnesses, which involves hospital admissions, multiple medications and its associated ADR’s [3]. Inappropriate use of medicines is one of the challenges of the public health domain and may lead to serious (ADRs) [4]that account for 3% to 23% of hospital admissions, prolong hospital stays, and increase in morbidity and mortality [5].

According to WHO, polypharmacy is defined as the concurrent use of five or more different prescription medications. Previous studies have provided evidence that the probability of ADRs among geriatric patients is estimated at 6% when two drugs are taken, increases to 50% when five drugs are taken, and becomes 100% when eight or more drugs are taken simultaneously [6]. Polypharmacy has also been documented as a major risk factor for ADRs in the developed countries [7]. Ageing has a strong impact on the pharmacokinetics and pharmacodynamics, comorbidity, and patterns of medication that may contribute to an increased risk of adverse events. A study from Malaysia found higher incidence of polypharmacy among geriatric inpatients (62.8%) on admission and it was associated with the high prevalence of cardiovascular diseases and diabetes mellitus [8].

Few epidemiological studies investigating the role of polypharmacy among the geriatrics have been almost exclusively conducted in the developed countries [9], [10]. However, there is a scarcity of evidence from Asian countries including Pakistan, and prospective studies investigating the association between polypharmacy and ADRs are almost lacking [11], [12]. Hence, determining the true incidence along with strength of association of ADRs due to polypharmacy among geriatrics would help in designing guidelines and policies for this vulnerable population. We hypothesize that the risk of ADRs among the exposed (polypharmacy) was greater than that of the un-exposed group. The overall aims of the study were to estimate the incidence of ADRs and its association with polypharmacy among the geriatric cohort attending outpatient clinics at a tertiary care center in Karachi, Pakistan.

## Methods

### Study Design and Population

**Figure 1:**
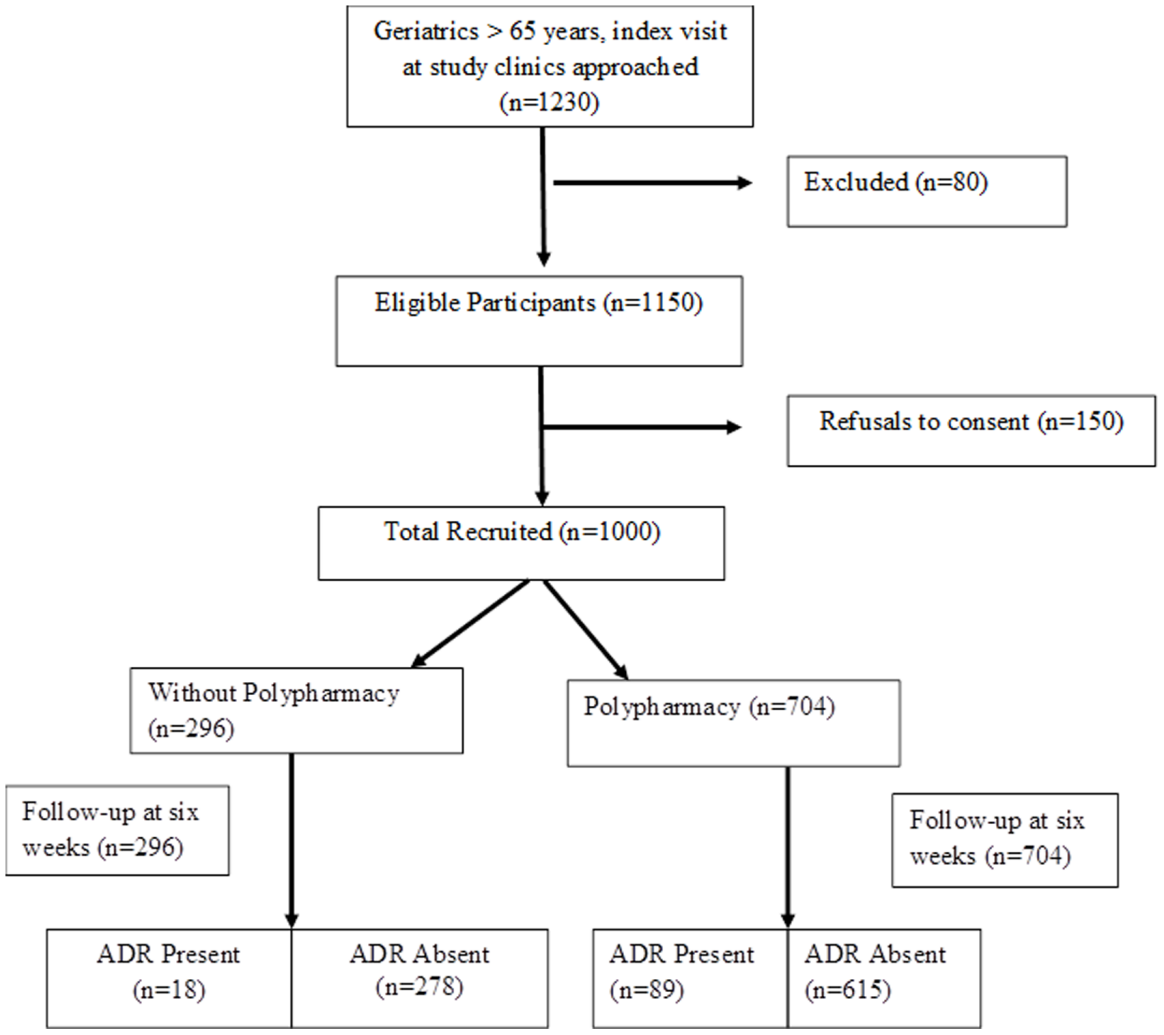
Flow of Study Participants.

A prospective cohort study was conducted during April 2012 to March 2013 at Aga Khan University Hospital (AKUH) Karachi, Pakistan, a 563 bedded, Join Commission International (JCI) accredited tertiary care center providing state of the art health care facilities. The study recruited a cohort of 1000 geriatrics patients having age ≥65 years, using consecutive sampling technique, either male or female at their index visit to any sub-specialty ambulatory care clinics (Family Medicine, Medicine, Surgery, Obstetrics and Gynecology) and was followed for six weeks to collect relevant factual data for the incidence of ADRs due to polypharmacy. **Figure 1** shows the flow of participant in the study. Subjects who were transferred to inpatient departments directly from clinic or those who required hospital admission or known to be mentally disabled or were suffering from advanced neurological diseases like dementia or acute confusion were not recruited. Written informed consent was obtained from each participant at the time of recruitment, those who were not able to read or write, detailed explanation was provided about the study protocol by the data collector and thumb impression was obtained in presence of a family member.

#### Ethics Statement

Ethical approval for this study was obtained from the ethical review committee of Aga Khan University (AKU FWA 00001177).

### Ascertainment of Polypharmacy

Trained interviewers screened and enrolled participants on the basis of standard definition of Polypharmacy which is defined by WHO as the, “concurrent use of five or more different prescription medications [13].” Multiple sources like computerized medical records, patient’s medical files and pharmacy prescriptions were used for data extraction to avoid any miss outs. The unexposed group was defined as patients receiving less than five drugs at their index visit. Telephonic interviews were conducted once weekly till the sixth week by an interviewer to follow the participants if they had developed any ADRs. At the index visit, interviewer collected complete information related to dug history. Subjects’ progress notes were also reviewed to determine any information related to the addition of new drugs, newly developed ADR’s, and critical laboratory values *(see Appendix S1).*

### Ascertainment of Adverse Drug Reaction

We obtained information on ADRs defined as “the presence of undesired outcomes due to receiving medicines” from multiple sources [14]. Two physicians and one pharmacist independently reviewed each reported ADR to determine the likelihood that the event was connected to a medication. A thorough literature search was performed before labeling any case as an ADR [15]. Besides this, the standard ADR Reporting Form by the drug and poison information center at the Department of Pharmacy Services, AKU, was also used to record all the essential information regarding the adverse effects: suspected drugs, suspected reaction, date of onset, date when the adverse effects ceased and severity of the ADR experienced (fatal, non-fatal). Subjects with ADRs were formally referred to their primary investigators.

### Drug characteristics by Anatomical Therapeutic Chemical

Drugs involved in the ADRs were coded into various drug classes according to Anatomical Therapeutic Chemical (ATC) classification based on WHO-ATC Index 2005 [16]. In the ATC classification system, the drugs are divided into different groups according to the organ or system on which they act and their chemical, pharmacological and therapeutic properties.

### Covariables

Covariables were collected from administrative questionnaires at baseline. Covariables included age (65–70, 71–80 and >80 years), sex, educational status (Can’t Read or Write, ≤5, 6–14, and >14 years of education), occupational status (unemployed/retired, employed), polypharmacy (no, yes), medication data with dose, frequency and duration, frequency of dose missing (no, yes) and non-prescription drugs (herbal, homeopathic, over the counter).

### Statistical Methods

The sample size was calculated by taking into account the objectives of the study. We used Epi Info Version 6 to calculate the sample size. The calculations were based on the assumption that polypharmacy among the Pakistani geriatrics is 50% (as we do not have any information regarding these in our community). By taking into account all of these figures together with 99% confidence interval, and exposed to non-exposed ratio of 1∶1 with 90% power and risk ratio of 1.3, the sample size came out to be n = 750. After adjusting 30% for non -responders the final required sample size was approximately 1000 geriatric participants.

In the analysis of cohort of 1000 geriatrics, we evaluated polypharmacy at baseline and cumulative incidence of ADRs, in relation to other baseline characteristics. The incidence of ADRs was calculated and Pearson chi-square tests were used to evaluate differences between categories. The Proportional hazard assumption was checked for all independent variables. A multivariable Cox regression analysis was used to study the independent association of variables with the presence of ADRs. The Relative Risk (RR) with 95% Confidence Intervals (CIs) was estimated. In the Cox model we adjusted for age, gender, employment status, use of nonprescription medications at baseline as potential confounding factors.

Statistical Package for Social Sciences (SPSS) Version 19 was used for analysis.

## Results

We followed 1000 elderly patients for six weeks after enrollment, and identified 107 (10.7%) ADRs in the full cohort. The overall occurrence of ADRs due to polypharmacy was 70%. Males were reported to have greater incidence of ADRs (59%). We found slightly higher risks of ADRs among population of illiterate persons when compared to people with higher level of education. The hazard ratios for different levels of education status ranged from 0.8–1.7. Likewise, use of non-prescription medicines, including herbal and homeopathic medicines, carries much higher risk of ADRs. All other characteristics were comparable between ADRs positive and ADRs negative groups as shown in *table 1*. Later, we compared subjects according to exposure status however; none of the characteristics were statistically different between the two groups as shown in table 2.

**Table 1:** Descriptive and Univariate Cox Analysis Along With 95% Confidence Intervals of Eligible Geriatrics Attending Tertiary Care Center.

Variable	ADR Negative	ADR Positive	*Unadjusted RR	95% CI
	n = 893	n = 107		
**Age**				
65–70	448 (50.2)	59 (55.1)	Reference	
71–80	359 (40.2)	42 (39.3)	1.8	0.77–4.13
Above 80	86 (9.6)	6 (5.6)	1.6	0.68–3.77
**Gender**				
Male	422 (47.3)	63 (58.9)	Reference	
Female	471 (52.7)	44 (41.1)	1.5	1.03–2.2
**Level of Education**				
More than 14 years	43 (4.8)	5 (4.7)	Reference	
Can’t Read Or Write	269 (30.1)	36 (33.6)	1.7	1.3–2.8
Less than 5 years	119 (13.3)	25 (23.4)	1.6	1.3–4.3
6–14 years	462 (51.7)	41 (38.3)	0.8	0.2–0.9
**Occupation**				
Employed	421 (47.1)	45 (42.1)	Reference	
Unemployed	472 (52.9)	62 (57.9)	1.2	0.8–1.7
**Use of Concurrent Homeopathic Medicine**				
No	877 (98.2)	94 (87.9)	Reference	
Yes	16 (1.8)	13 (12.1)	4.6	2.5–8.2
**Use of Concurrent Herbal Medicine**				
No	873 (97.8)	95 (88.8)	Reference	
Yes	20 (2.2)	12 (11.2)	3.8	2.1–6.9
**Poly-Pharmacy**				
No	278 (31.1)	18 (16.8)	Reference	
Yes	615 (38.9)	89 (83.2)	2.1	1.2–3.4

*Relative risk obtained from Cox regression analysis.

**Table 2:** Characteristics of Eligible Participants Attending Tertiary Care Center, According To Exposure Status.

Variable	Polypharmacy (n) %	
	Yes	No
**Age**		
65–70	345 (49)	162 (54.7)
71–80	286 (40.6)	115 (38.9)
Above 80	73 (10.4)	19 (6.4)
**Level of Education**		
More than 14 years	35 (5)	13 (4.4)
Can’t Read or Write	221 (31.4)	84 (28.4)
Less than 5 years	103 (14.6)	41 (13.9)
6–14 years	345 (49)	158 (53.4)
**Gender**		
Male	365 (51.8)	150 (50.7)
Female	339 (48.2)	146 (49.3)
**Use of Concurrent Herbal Medicine**		
No	686 (97.4)	282 (95.3)
Yes	18 (2.6)	14 (4.7)
**Use of Concurrent Homeopathic Medicine**		
No	691(98.2)	280 (94.6)
Yes	13 (1.8)	16 (5.4)

After adjusting for age, gender and occupational status, an adjusted multivariable model indicated that polypharmacy, low level of education and use of concurrent homeopathic medicines were significantly associated with ADRs among geriatrics. Risk of ADRs among elderly patients with polypharmacy was 2.3 (95% CI: 1.4–3.9) higher than those who took lesser number of medicines. Low level of education (i.e. those who were unable to read or write and those with less education than primary schooling) was more likely to be associated with ADRs. However, as the level of education increases the association with ADRs turns out to be protective (RR = 0.7, 95% CI: 0.5–0.9). Risk of ADRs among geriatrics who took concurrent homeopathic medicines was higher compared to those who did not (RR = 7.4, 95% CI 3.2–8.8), as shown in *table 3*.

**Table 3:** Adjusted Multivariable Analysis Showing Relative Risk of Adverse Drugs Reactions Along With 95% CI.

Variables	Relative Risk	95% CI
**Polypharmacy**		
No	*Reference*	
Yes	2.3	1.4–3.9
**Level of Education**		
More than 14 years	*Reference*	
Can’t Read Or Write	1.5	1.2–2.8
Less than 5 years	1.3	1.1–2.9
6–14 years	0.7	0.5–0.9
**Use of Concurrent Homeopathic Medicine**		
No	*Reference*	
Yes	7.4	3.2–8.8

In table 4, we calculated the incidence of ADRs according to the exposure to various drug classes. The highest incidence was found for antitussives and anti-dopaminergic drugs in our study group.

**Table 4:** Incidence of Adverse Drug Reactions According To Drug Exposure.

Drugs Classification	ADR negative	ADR Positive	Incidence per 1000 population
Antitussives	1	2	666
Anti-dopaminergic	1	1	500
Antipsychotics	4	2	333
Anti-hypertensive	68	10	128
Antibiotics	98	14	125
Supplements	74	10	119
Cardiovascular	491	57	104
Anti-diabetics	73	6	75
Anti-lipidemic	13	1	71
NSAIDs	18	1	52
Anti-peptic ulcer	42	2	45
Statins	2	0	0
Anticoagulants	2	0	0

## Discussion

In this prospective cohort study the incidence of ADRs with polypharmacy was found to be 10.5%, moreover, we also found high rates of polypharmacy in geriatric outpatients which is consistent with previous researches. In the current study, 68.2% of the elderly patients were taking more than five medications a day. This is slightly higher than the rates previously reported for geriatric population. We observed a statistically significant association between low levels of education and the concurrent use of non-prescription medicines both before and after adjusting for potential confounders. With the hope of directing intervention efforts; many associations have been proposed for ADRs among the geriatric population (aged 65 years or older) since they are mostly prescribed with multiple medications which make them vulnerable to ADRs.

Investigators suggest that longer stay in hospital is one of the probable cause for the occurrence of ADRs in geriatrics and it is defined as an undesirable condition caused by the use of multiple medications [17].

The incidence of 10.6% ADRs found in this study is low as compared to other general or outpatient studies conducted in different countries [8], [9], [18]. This could be due to the difference in the methodological aspects of the study particularly the study population and the self-reporting of ADRs in the follow-ups which was conducted through telephonic calls in the current study. This probably implies an underestimation of actual occurrence of adverse effects. Another reason for the lower frequency of ADRs observed in our study is probably due to the method of extracting information on the use of complementary and alternative medicines (herbal 3.2% and homeopathic 2.9%) which is missing in most of the studies recording ADRs along with poly-pharmacy. Interestingly, among these patients who were taking CAM, the occurrence of ADRs were similar i.e. 12%. Most studies conclude that most of the ADRs in outpatients turn out to be harmless; however this study opposes this fact where 13% patients (n = 14 out of 107) had to make a hospital visit as a result of ADRs [19].

The use of CAM i.e. herbal and homeopathic has become increasingly popular in both developed and the developing countries [20], [21]. In this study, 14% of the elderly patients were using homeopathic and 13% were using herbal medications. This is lower than the rates reported in other studies where the use of CAM is as high as 66% [21]. One of the strong motivational factors to use CAM is their perceived remedial benefits, and safety profile. However, we can only speculate about the role and benefits of CAM in certain diseases as the role of CAM in chronic diseases is still controversial. In the current study those elderly who were taking concurrent homeopathic medication were 7 times more at risk of developing ADRs. The probable reason for this high risk of developing ADRs can be due to the fact that many commonly used CAM products have the potential to interfere with the intended action of concomitant prescription medications, which could lead to serious drug interactions and in turn increase the risk of ADRs. Nevertheless, it is important to educate the patients about the risks and benefits of CAM. Studies are required to determine the impacts of CAM, particularly its impact when used in conjunction with prescribed medicines.

It is evident that some drugs such as anticholinergic and antipsychotics can impair the physical and cognitive function in the elderly patients [22]. In the current study antipsychotics had an ADR incidence of 333 per 1000 population and anti-hypertensive had incidence of 128/1000. This implies that the more drugs with these effects that the elderly patients are exposed to (number and dose), the poorer will be their quality of life and they will be more prone to ADRs, as evident from the results of the current study that those elderly patients who were positive of polypharmacy had 2.3 times more risk of developing ADRs [23].

There are several strengths of our study; a cohort study design was carefully chosen which is ideal in predicting the causal association of exposure with the outcome so inferences can be drawn regarding causality of association between polypharmacy and other factors with the ADRs. We collected data from OPD prescriptions to avoid any miss outs. In addition, we estimated the incidence of ADRs due to polypharmacy, defined as the use of >5 scheduled medications in the line of WHO guidelines. The criteria of more than five medications used included only systemic and routinely administered medications. Moreover, there was no loss in following-up in our cohort. However, our study has certain limitations that need to be considered while interpreting the results. This was a hospital based study hence generalizability to public sector settings remain questionable. We followed our subjects for a duration of six weeks only, thus adverse effects arising after this time may not be captured and this might have underestimated our results.

To the best of our knowledge, this is the first Asian study to record the incidence of ADRs in geriatric outpatients with polypharmacy; our study confirms the notion that elderly patients are more likely to experience these adverse reactions as the result of age-related increase in the frequency of drug use, sensitivity to drug effects, and prevalence of predisposing conditions that can increase the frequency and severity of ADRs.

With the current state of health system utilization and health-seeking behavior in Pakistan, it is highly desirable to reduce the divergence by exploring more opportunities for integration of patient safety. As a way forward this study and its findings may encourage the physicians to implement judicious prescribing.

Appropriate educational, managerial or regulatory strategies are needed for evidence based prescribing. It is also important that medications for the elderly patients be reviewed periodically for indication, therapeutic aims, dose, efficacy and probable side effects. Moreover, the benefit and risks of treatment (drugs) including the impact on functions and quality of life should be discussed with patients and their caregivers.

In conclusion, in this reasonably large hospital based prospective cohort study of geriatrics, the incidence of ADRs due to poly-pharmacy is high. Several factors including low level of education and use of non-prescription medications remain responsible for the high burden. While additional research with more sophisticated design is needed to confirm our findings, our data suggests that a comprehensive strategy for evidence based prescribing must be implemented.

## Research in Context

Stimulated by JK. Nguyen’s findings [9], we performed a hospital based prospective cohort study where we surveyed the geriatric population (>65 years) attending the outpatient clinics of Aga Khan University Hospital Karachi throughout the year 2012. The data was collected from different consulting clinics of Anesthesia, Family Medicine, Internal Medicine and Obstetrics. The sampled geriatric population was recruited from clinics and followed to collect relevant factual data for the incidence of adverse drug reactions due to polypharmacy. We used the operational definition for polypharmacy as the concurrent use of five or more different prescription medications.

We initiated the data collection by gathering demographic information where 30% of the total patients were not able to read or write and 53% of the patients were unemployed. The mean age was found to be 70 years (range 65–70). Later on, during the follow up phase, the patients were asked about self-medication and its frequency, 53% patients were self-medicating via OTC drugs. On asking whether a pharmacist provided them any valuable information regarding side/adverse effects of the dispensed drug, 35% patients reported a negative response. The overall occurrence of polypharmacy was 68% while the incidence of ADRs along with polypharmacy was found to be 10.5%. About 3.2% of the participants relied on herbal medicines and 3% on homeopathic medicines.

Our study supports the findings of JK. Nguyen and colleagues. Our Cox adjusted model shows that polypharmacy was 2.3 times more associated with ADRs. Con-current homeopathic use was 7.4 times and those who were unable to read and write were 1.5 times more at risk of developing ADRs.

## Supporting Information

Appendix S1 (PDF)

Data S1 (SAV)
