# Supplementary material for: Effects of Polypharmacy on Adverse Drug Reactions among Geriatric Outpatients at a Tertiary Care Hospital in Karachi: A Prospective Cohort Study
Source: PLoS One. 2014 Nov 17;9(11):e112133. doi: 10.1371/journal.pone.0112133 (PMC4234513; doi:10.1371/journal.pone.0112133)
Supplement: Appendix S1 — (PDF) [file pone.0112133.s001.pdf]

FORM 1 BASE LINE INFORMATION

**Identifier:**		**MR NO**	
Clinic Number		Interviewer name	
Date of interview			
Time of interview:<br>**Start___________ am/ pm end __________ am/ pm**			

No.	Questions	Codes	Skip	Responses
1.	Name of Participant			-------------------------
2.	Gender	a) Male<br>b) Female		
3.	Residential Address			
4.	Telephone (very important)			Residential:--------------<br>Mobile:---------------
5.	Who is your primary care giver?			Name:______________________<br>Relation:__________________<br>Mobile:____________________<br>Best available time for interview
6.	Age (years)			________________
7.	Height in feet			
8.	Weight in Kg			
9.	Temperature			
10.	What is your educational status?	a) Cannot read and write<br>b) Primary<br>c) Middle<br>d) Matriculate<br>e) Intermediate<br>f) Graduate<br>g) Post graduate		
11.	Occupation	a) Housewife/ not employed<br>b) Govt. servant<br>c) Armed forces<br>d) Business<br>e) Teacher<br>f) Private firm<br>g) Domestic		

		servant<br>h) Student<br>i) Self-employed		
12.	What is the current diagnosis? (check from file and write)			
13.	Other co-morbidities			
14.	Previous medications	a) No previous medications<br>b) ------------------<br>c) ------------------<br>d) ------------------<br>e) ------------------	if a skip to	
15.	Previous Medications Dose	a) ------------------<br>b) ------------------<br>c) ------------------<br>d) -----------------<br>e) -----------------		
16.	Previous Medications Duration			
17.	Current Medications	a) -----------------<br>b) -----------------<br>c) -----------------<br>d) ----------------<br>e) -----------------<br>f) ------------------<br>g) -----------------<br>h) ------------------		
18.	Current Medications Dosage			

19.	Current Medications Duration			
20.	Is this Polypharmacy?<br>(RMO will review the profile and label patient as yes or no)	a) Yes<br>b) No		
21.	Name of Physician patient registered with?			
22.	Are you currently taking any herbal medications?	a) Yes<br>b) No		
23.	Are you currently taking any homeopathic medications?	a) Yes<br>b) No		

Follow-up Form

ID NO		**MR NO**	
Clinic Number		Interviewer name	
Date of interview			
Time of interview:			

**Start___________ am/ pm End __________ am/ pm**

My name is ____________ and I am a research medical officer for the study you consented to yesterday. . You are participating in a study to improve the way medications are prescribed.

Is this a good time for you to talk?

If not - - - - When would be a good time for us to call you?

This interview will last approximately 15 minutes. Would you like to participate?

If not - - - - Thank you very much for your time.

Patients sometimes experience problems due to their medications. We want to better understand how often they occur and how they can be prevented.

We are interviewing patients who have been prescribed medication by their doctor. This interview will start off by asking you for some basic health information. I will then ask you some specific questions about the medications you have been prescribed and about any non-prescription medications that you use regularly.

Let me assure you that that the information you provide is completely confidential (as previously mentioned in the consent as well). Your participation is voluntary and you may skip any questions that you do not feel comfortable answering. Also, you may stop the interview at any time.

In order to use our time most effectively, would you please take a few moments now to collect in front of you all the medications and dietary or herbal supplements which you take on a regular basis, both prescription and non-prescription.

1	Did you fill your Prescription?	No<br><br>Yes (skip to question #7)
2	If no- why not (stop - do not continue with follow-up)	1 No time, too busy<br>2 Still have some old medications left<br>3 Afraid to take medication<br>4 Couldn't afford medication<br>5 Not covered by insurance<br>6 Don't need it<br>7 Other ____________________________
3	When did you fill this prescription?	__ __/ __ __/ __ __
4	Please read the directions for use directly from the medication bottle.	____<br>____<br><br>____
5	What was the total quantity dispensed to you? Please read any precautions or warnings from the bottle.	Quantity dispensed:<br><br>____<br>_________<br><br>____<br>_________<br><br>Precautions/Warnings:<br><br>____<br>_________<br><br>____<br>_________<br><br>____<br>_________<br><br>____

	________

The next few questions ask you about information you received when you were given your prescription.

6 When you received your prescription, did your doctor tell you what the medication was for?	1 No<br>2 Yes<br>3 Don't know
7 Did your doctor tell you about the possible side effects?	1 No<br>2 Yes<br>3 Don't know
8 When you got your prescription filled, did your pharmacist offer you verbal counseling?	1 No (skip to #14)<br>2 Yes<br>3 Don't know
9 Did you accept verbal counseling from the pharmacist?	1 No<br>2 Yes (skip to question #15)
10 If no, why not?<br>(answer question and then skip to question #16)	Was not offered to me:<br>1. I already got information from my MD<br>2. Medication was a refill-got information from my pharmacist before<br>3. I waited long enough and did not have enough time<br>4. I didn't think I needed any<br>5. Other ____

11 Specifically, did the pharmacist tell you what the medication was for?	1 No<br>2 Yes<br>3 Don't know/remember
12 Did the pharmacist tell you about possible side effects?	1 No<br>2 Yes<br>3 Don't know/remember
13 Did you get printed information about this medication from your pharmacist?	1 No<br>2 Yes<br>3 Don't know/remember

14 How many prescription bottles do you have in front of you? ________
Would you please answer the following questions by reading directly from your medication bottles.

Medication Name	Type of prescription			What do you take this medication for?		
	New	Refill	DK	DK	K	If K, specify
1-						
2-						
3-						
4-						
5-						
6-						
7-						
8-						
9-						
10-						
11-						
12-						

13-						
14-						
15-						
16-						
17-						

K= Know DK= Don't Know

15 Generally do you take **all** the doses of your medications as prescribed by your doctor?	1 No (specify medication)____________<br>2 Yes<br>3 Don't know<br>4 Refuse to answer<br>5 Not applicable
16 What do you usually do if you miss a dose of medication?	1 Take an extra dose<br>2 Skip that dose<br>3 It varies with the medication<br>4 Don't know/remember<br>5 Never miss a dose (skip to question #23)<br>6 Take as soon as I remember
17 In the last week, how many doses do you think you have missed?	1 None<br>2 One<br>3 Two<br>4 Three<br>5 Four<br>6 Five<br>7 Six or more
18 Why did you miss these doses? (multiples may be checked)	1 Forgot to take medication<br>2 Ran out of medication<br>3 Medication not available (misplaced or not with patient at time of dose)<br>4 Felt that medication was not needed<br>5 Side effect of medication<br>6 Other __________________________
19 In the last week, how many additional doses do you think you have taken?	1 None (stop - skip to question #24)<br>2 One<br>3 Two<br>4 Three<br>5 Four<br>6 Five<br>7 Six or more
20 Why were the extra doses taken?	1 Tried to catch up<br>2 Thought it was better to take more<br>3 Forgot that medication was already taken<br>4 Other ___________________________

Now we're going to talk about any non-prescription medications which you use.

21 Do you take any non-prescription drugs (herbal supplements, over-the counter drugs, dietary supplements)on a regular basis?

1 No (do not as question 25)
2 Yes

The following questions will ask you the names, strengths and reasons for use of all of the non-prescription medications you use regularly. Again, please answer by reading directly from your medication bottles. How many bottles do you have in front of you? ________

22 Non-prescription Medication list:

**Medication Name and Strength**	**How frequently do you take this medication?**<br>■ daily<br>■ weekly<br>■ monthly	**What do you take this medication for?**<br><br>DK - don't know	**Have you had any problems with this medication?**		
			No	Yes	If yes, specify
1-					
2-					
3-					
4-					

5-					
6-					
7-					

24. I am now going to begin by asking you several questions about your health during the past three months.

In the past three months have you had any of the following problems.

	A	B	C	D	E	F	G
	**General Symptoms**<br><br>**1= No**<br>**2= Yes**<br>**3= Refuse/ DK**<br><br>***If no, then STOP***	**How long ago did this symptom start?**<br><br>**1=<1 day**<br>**2= 1-3 days**<br>**3= 4-7 days**<br>**4= 7-28 days**<br>**5= 1-3 months**<br>**6= > 3 months**	**Is this related to Medication?**<br><br>**1= No**<br>**2= Yes (specify)**<br>**3= Yes, target rx (specify)**<br>**4= DK**<br><br>***If no, then STOP***	**Are you still taking the medication?**<br><br>**1= No**<br>**2= Yes**	**Do / did this symptom occur with every dose?**<br><br>**1= No**<br>**2= Yes**<br>**3= DK**	**How soon after taking the medication did these symptoms occur?**<br><br>**1=<1 day**<br>**2= 1-3 days**<br>**3= 4-7 days**<br>**4= >7 days**	**Have /did y discuss(ed) symptom w your docto**<br><br>**1= No**<br>**2= Yes**<br><br>***If no, skip to***
24.1 Problems with sleep							
24.2 Changes in mood							
24.3 Gastrointestinal /stomach difficulties							
24.4 Dizziness or problems with balance							
24.5 Headache							
24.6 Fatigue							
24.7 Muscular aches							

24.8 Incontinence or trouble holding your urine							
24.9 Problems with sexual function							
24.10 Skin rash or itching							
24.11 Other ___________							
